# The effect of synchronized pauses on the coding strategies of cerebellar nuclear neurons: a modeling study

**DOI:** 10.1186/1471-2202-16-S1-P241

**Published:** 2015-12-18

**Authors:** Shyam Kumar, Benjamin Torben-Nielsen, Erik De Schutter

**Affiliations:** 1 Computational Neuroscience Unit, Okinawa Institute of Science and Technology, Onna-son, Okinawa, Japan; 2 Department of Theoretical Neurobiology and Neuroengineering, University of Antwerp, Wilrijk, Belgium

## 

Purkinje cells (PC) of the cerebellar cortex have two distinct firing signatures: simple and complex spikes. The simple spikes are due to the intrinsic mechanisms of the cell and synaptic inputs coming through the parallel fibers and molecular layer interneurons [1-3]. The PCs also emit complex spikes, which are due to strong excitation coming from the axons of the inferior olive cells, the climbing fibers [2]. The simple spikes are highly regular intertwined with short pauses [4] while complex spikes occur sporadically and consist of burst of spikelets [5-7]. How can the cerebellar nuclei neurons downstream of the PCs make sense of this complex firing pattern?

In this study we analyze how PC synchrony in the context of "pauses" in simple spikes affects different coding mechanisms of the neurons of the cerebellar nuclei. To this end, we use a computational model of a cerebellar nuclear neuron [8] and synthetic PC spike trains. The coding mechanisms of cerebellar nuclear neuron can be broadly categorized as rate [9,10] and time coding [11]. We define PC synchrony as synchronized Purkinje neuron pauses with either pause beginning or pause ending spikes precisely synchronized [4]. With varying amount of pause synchrony for the above mentioned synchrony types, we analyzed its effects on time locking and rate coding in a nuclear cell.

We find that both the amount and type of pause synchrony is encoded as rate increases in the firing of the nuclear cell. Synchronized pauses briefly release the nuclear neuron from inhibition giving rise to well-timed spikes. Further, pauses synchronized with pause ending spikes caused greater firing rate modulation in the nuclear cell while pause beginning type synchrony enhanced the degree of timelocking. We also analyze the effect of pause length and spike jitter on the timelocking phenomenon of the nuclear neuron. We argue that these results lead to better understanding of how PC pause synchrony is processed in its target nuclear neuron.

## References

[B1] Raman IM Bean BP Ionic Currents Underlying Spontaneous Action Potentials in Isolated Cerebellar Purkinje Neurons J Neurosci 1999 19 5 1663 1674 10024353 10.1523/JNEUROSCI.19-05-01663.1999 PMC6782167

[B2] Palay SL Chan-Palay V Cerebellar Cortex 1974

[B3] Gundappa-sulur G Schutter EDE Bower JM Ascending Granule Cell Axon: An Important Component of Cerebellar Cortical Circuitry 1999 408 580 596 10.1002/(sici)1096-9861(19990614)408:4<580::aid-cne11>3.0.co;2-o 10340507

[B4] Shin SL De Schutter E Dynamic synchronization of Purkinje cell simple spikes Journal of Neurophysiology 2006 96 6 3485 3491 16987931 10.1152/jn.00570.2006

[B5] Eccles JC Llinás R Sasaki K The excitatory synaptic action of climbing fibres on the Purkinje cells of the cerebellum J Physiol 1966 182 2 268 296 5944665 10.1113/jphysiol.1966.sp007824 PMC1357472

[B6] Llinás R Sugimori M Electrophysiological properties of in vitro Purkinje cell dendrites in mammalian cerebellar slices J Physiol 1980 305 197 213 7441553 10.1113/jphysiol.1980.sp013358 PMC1282967

[B7] Shin SL Rotter S Aertsen A De Schutter E Stochastic description of complex and simple spike firing in cerebellar Purkinje cells The European Journal of Neuroscience 2007 25 3 785 794 17328774 10.1111/j.1460-9568.2007.05308.x

[B8] Steuber V Schultheiss NW Silver RA De Schutter E Jaeger D Determinants of synaptic integration and heterogeneity in rebound firing explored with data-driven models of deep cerebellar nucleus cells Journal of Computational Neuroscience 2011 30 3 633 658 21052805 10.1007/s10827-010-0282-z PMC3108018

[B9] Armstrong BYDM Rawson JA Activity patterns of cerebellar cortical neurones and climbing fibre afferents in the awake cat 1979 289 425-448 10.1113/jphysiol.1979.sp012745 PMC1281378 458677

[B10] McDevitt CJ Ebner TJ Bloedel JR Relationships between simultaneously recorded Purkinje cells and nuclear neurons Brain Research 1987 425 1 1 13 3427412 10.1016/0006-8993(87)90477-x

[B11] Person AL Raman IM Purkinje neuron synchrony elicits time-locked spiking in the cerebellar nuclei Nature 2012 481 7382 502 505 22198670 10.1038/nature10732 PMC3268051

